# ﻿*Primula xinjingensis* (Primulaceae), a new species from Guizhou, China

**DOI:** 10.3897/phytokeys.265.168043

**Published:** 2025-11-04

**Authors:** Sheng-Hu Tang, Ze-Xu Long, Fang-Wen Li

**Affiliations:** 1 Gesneriad Conservation Center of China (Guizhou), National Forestry and Grassland Administration Key Laboratory for Biodiversity Conservation in Karst Terrain of Southwestern China, Guizhou Botanical Garden, Guiyang 550000, China Guizhou Botanical Garden Guiyang China; 2 School of Biological Science, Guizhou Educational University, Guiyang 550000, China Guizhou Educational University Guiyang China; 3 Chengdu Botanical Garden (Chengdu Park City Botanical Science Research Institute), Chengdu 610000, China Chengdu Botanical Garden Chengdu China

**Keywords:** karst flora, new taxon, *
Primula
*, Yanhe County

## Abstract

A new species, named *Primula xinjingensis*, has been described from north-eastern Guizhou, China. It belongs to the sect. Monocarpicae. The species resembles *P. pellucida* in having efarinose plants, the shape of the leaf blade, and recurved calyx lobes in fruit. However, it differs from *P. pellucida* by possessing a campanulate calyx, lanceolate calyx lobes, narrowly cuneate-obovate corolla limb lobes, and a style reaching the throat in the pin flowers. It also shares similarities with *P. divaricata* and *P. epilithica* in terms of efarinose plants and the shape of the leaf blade. Nevertheless, it is distinct from these two species by the absence of rhizomes, having recurved calyx lobes in fruit, lanceolate calyx lobes, and narrowly cuneate-obovate corolla limb lobes. The new taxon has been assessed as “Data Deficient” (DD) according to the IUCN standards.

## ﻿Introduction

*Primula* L. is a large genus belonging to the family Primulaceae, comprising approximately 548 species worldwide (POWO 2025). This genus primarily occurs in the temperate and alpine regions of the Northern Hemisphere (Hu 1990; Hu and Kelso 1996). In China, approximately 351 species have been documented (Liu 2025). In recent years, numerous new taxa have been discovered in China (IPNI 2025), including *P. yanbianensis* T.Shuai, Lei Cai & Z.K.Wu (Shuai et al. 2025), *P. medogensis* W.B.Ju, Bo Xu & X.F.Gao (Ju et al. 2023), *P. zhengyii* Bin Yang & Y.H.Tan (Yang et al. 2023), and *P. xinningensis* Wei Zhang bis & J.W.Shao (Zhang et al. 2022).

The section Monocarpicae Franch. ex Pax of the genus *Primula* is characterized by plants having multicellular hairs, membranous leaves with a distinct petiole and a round or heart-shaped blade base, a campanulate calyx, and a globose or cylindrical capsule dehiscing by valves (Hu 1990). This section is a small group consisting of 21 species in China (Hu and Kelso 1996; Xu et al. 2016a, 2016b, 2017, 2022).

During a field survey conducted in March 2025 in Yanhe County, Guizhou Province, China, we discovered some flowering plants belonging to the genus *Primula*. In April 2025, we returned to collect specimens and take photographs. In June 2025, we harvested the capsules from the plants cultivated at the Guizhou Botanical Garden. The plants are characterized as perennial, efarinose herbs lacking rhizomes. They feature a 5–9-lobulate leaf blade with multicellular hairs, lanceolate calyx lobes that are recurved in fruits, and narrowly cuneate-obovate corolla lobes. After thorough comparisons, we concluded that they represent a new species belonging to the section Monocarpicae.

## ﻿Materials and methods

The morphological characteristics of approximately 100 mature individuals were observed, and 20 selected flowers were carefully observed and measured in the field. A microscope (Olympus SZ61, Tokyo, Japan) was used for micro-observation. The plant was described following the terminology used by Hu and Kelso (1996). All the species belonging to the section Monocarpicae in China have been examined using type specimens or the descriptions provided by Hu (1990) and Hu and Kelso (1996). Relevant literature was consulted, including Xu et al. (2016a, 2016b, 2017, 2022). The relevant images were sourced from virtual herbaria and databases, including E (https://data.rbge.org.uk/search/herbarium/), P (https://science.mnhn.fr/all/search), iPlant (http://www.iplant.cn/), and CVH (https://www.cvh.ac.cn/), CFH (https://www.cfh.ac.cn/) and CUBG (https://image.cubg.cn/).

### ﻿Taxonomic treatment

#### ﻿ Primula xinjingensis

Taxon classificationPlantaeEricalesPrimulaceae

Sheng H.Tang & F.W.Li sp. nov.

90FC766F-A361-5221-A1D0-DE24969D4202

urn:lsid:ipni.org:names:77371343-1

Figs 1, 2, 3

##### Diagnosis.

The new species bears a striking resemblance to *Primula pellucida* Franch. in terms of the efarinose plants, the shape of the leaf blade, and the recurved calyx lobes in fruit. However, it is distinguished by having a campanulate (vs. broadly campanulate) calyx, lanceolate (triangular to ovate) calyx lobes, narrowly cuneate-obovate (vs. obovate) corolla limb lobes and a style reaching the throat (vs. exserted from the corolla tube) in the pin flowers. The new species also shares similarities with *P. divaricata* F. H.Chen & C.M.Hu and *P. epilithica* F.H.Chen & C.M.Hu in terms of leaf blade size and shape, as well as the campanulate calyx. Nevertheless, it is set apart from these two species by the absence of rhizomes (vs. their presence), lanceolate (vs. triangular or ovate-triangular) calyx lobes that are recurved (vs. erect) in fruits, and narrowly cuneate-obovate (vs. cuneate-obovate or obovate) corolla limb lobes.

**Figure 1.**
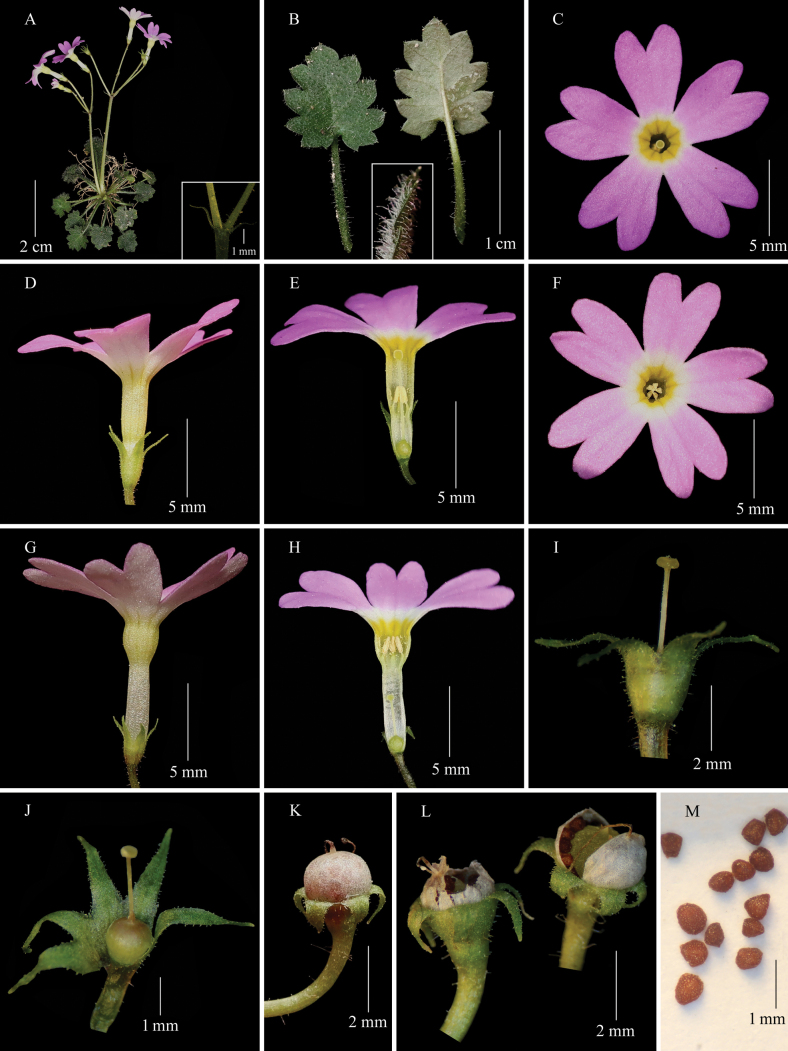
*Primula xinjingensis* sp. nov. A. Flowering plant, and bracts (inset); B. Adaxial and abaxial surfaces of the leaf blade, and multicellular hairs on both sides (inset); C, D, E. Pin flower; F, G, H, I, J. Thrum flower; K, L. Capsules; M. Seeds. Photographs by Sheng-Hu Tang.

##### Type.

China. Guizhou Province: Yanhe County, Xinjing Town, 28°52'N, 108°18'E, ca. 700 m, 3 April 2025, *Sheng H.Tang & Da-Zhu Tang 202504001* (holotype: CSH! [accession number CSH0220070]; isotype: the Guizhou Botanical Garden!).

**Figure 2.**
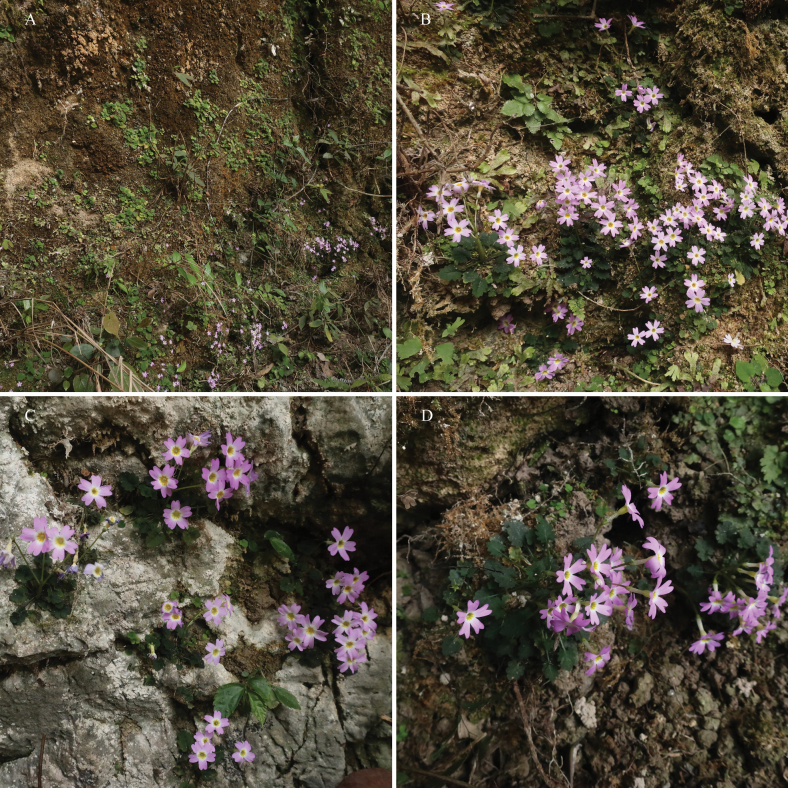
Habitats of *Primula xinjingensis* sp. nov. (Population at the type locality). Photographs by Sheng-Hu Tang.

##### Description.

Herbs perennial, efarinose, without rhizomes, up to 9 cm tall at anthesis. ***Roots*** numerous, fibrous. ***Leaves*** 7–20, forming a rosette; petiole 5–13 mm long, short-stalked glandular and sparsely multicellular hairs; leaf blade ovate or suborbicular, 4–12 × 4–10 mm, membranous when dry, abaxially and adaxially with multicellular hairs, base cordate, apex acute, margin 5–9-lobulate; lobules ovate to broadly ovate, margin entire, sometimes with 1 or 2 teeth, apex acute; lateral veins 3–4 pairs, obscure adaxially and prominent abaxially. ***Scapes*** 1–3, short-stalked glandular and sparsely multicellular hairs, 1–4 cm tall, umbel 1, rarely 2, 1–4-flowered; bracts 3, sometimes 2 or 4, linear-lanceolate, 2–3 mm long, short-stalked glandular on both sides. ***Flowers*** heterostylous. Pedicel 1–2 cm long, short-stalked glandular and sparsely multicellular hairs. Calyx campanulate, 2–4 mm long, slightly enlarged in fruit, short-stalked glandular and sparsely multicellular hairs outside, glabrous inside, parted to middle or slightly below; lobes 1.5–2.5 × 0.8–0.9 mm, lanceolate, recurved at anthesis sometimes, and recurved in fruit, apex acuminate. Corolla rose-purple; tube 6–9 mm long; limb 9–14 mm wide; lobes 4–6 mm long, narrowly cuneate-obovate, deeply emarginate. ***Pin flowers***: stamens 2–2.2 mm above base of corolla tube, style 4.5–7 mm long, reaching throat. ***Thrum flowers***: stamens toward apex of corolla tube, style 2.5–3 mm long. ***Capsule*** globose, 1.7–2.1 mm in diam., dehiscing by 2 valves or crumbling irregularly. Seeds numerous, ovoid or irregularly ovoid, 0.5–0.8 mm long, brown, testa reticulate.

**Figure 3.**
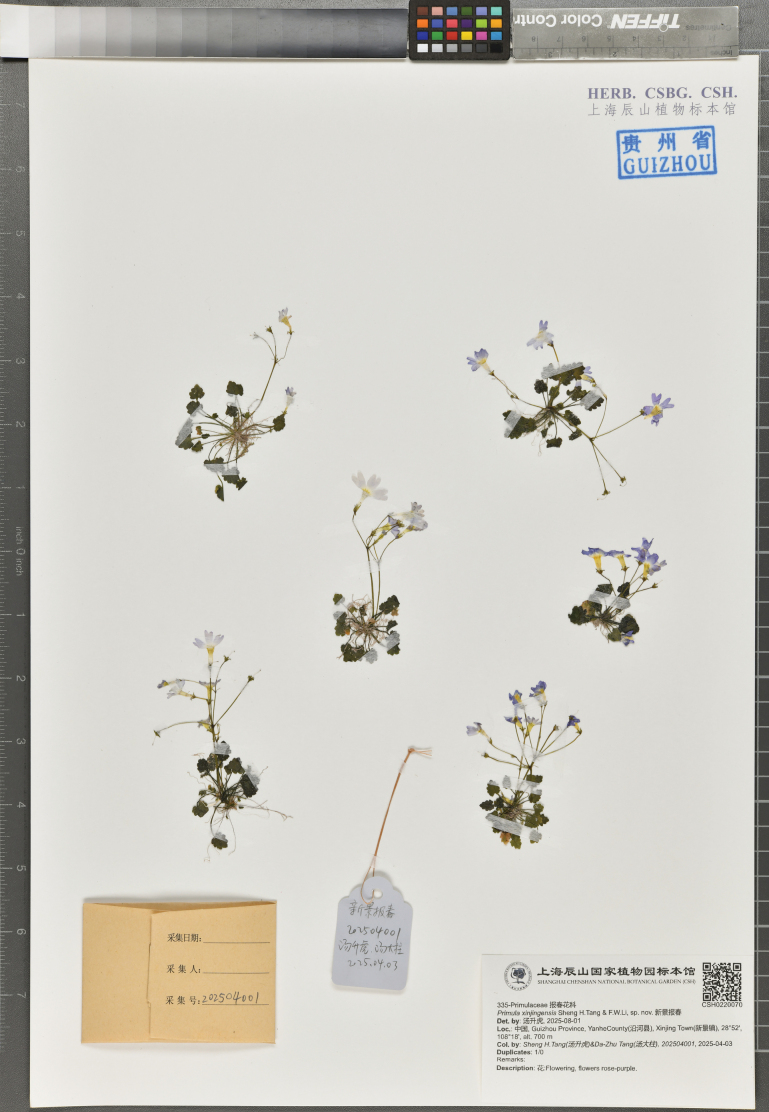
Holotype of *Primula xinjingensis* sp. nov. stored in CSH (*Sheng H.Tang & Da-Zhu Tang 202504001*, CSH0220070).

##### Phenology.

Flowering occurs from late March to early April, and fruiting occurs from May to June.

##### Etymology.

The new taxon was named for its locality, Xinjing Town, Yanhe County, Guizhou Province, China.

##### Vernacular name.

The Chinese name is “Xīn Jǐng Bào Chūn” (新景报春).

##### Distribution and habitat.

**Figure 4.**
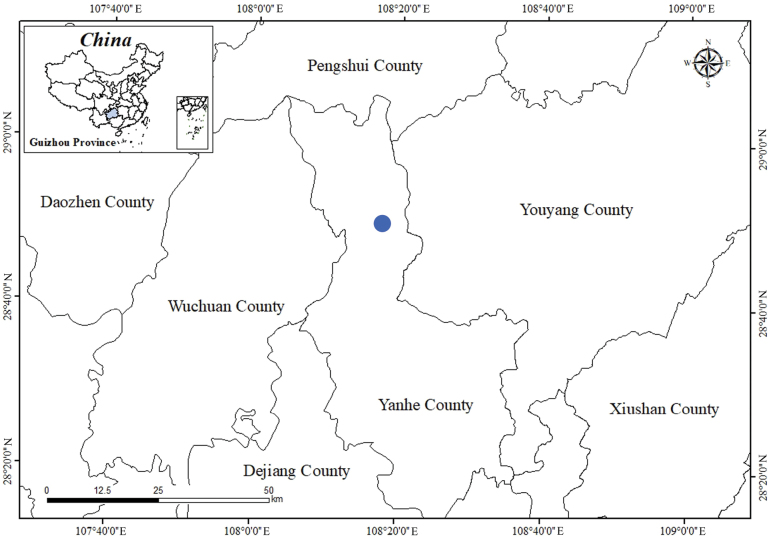
Location of the population of *Primula xinjingensis* sp. nov. in Yanhe County, Guizhou Province, China (indicated by the blue circle).

Only one population was discovered in Xinjing Town, Yanhe County, Guizhou Province, China (Fig. 4). It thrives on moist, shady cliffs and limestone surfaces. The primary companion species are *Androsace kouytchensis* Bonati, *Petrocodon scopulorum* (Chun) Yin Z.Wang, and *Ophiorrhiza chinensis* H.S.Lo.

##### Conservation status.

Only one population, consisting of approximately 200 mature individuals, was discovered at the type locality. Additional populations likely exist in this area. Until further investigation is conducted, the species should be designated as “Data Deficient” (DD) in accordance with the IUCN Red List Criteria (IUCN 2024).

##### Notes.

**Figure 5.**
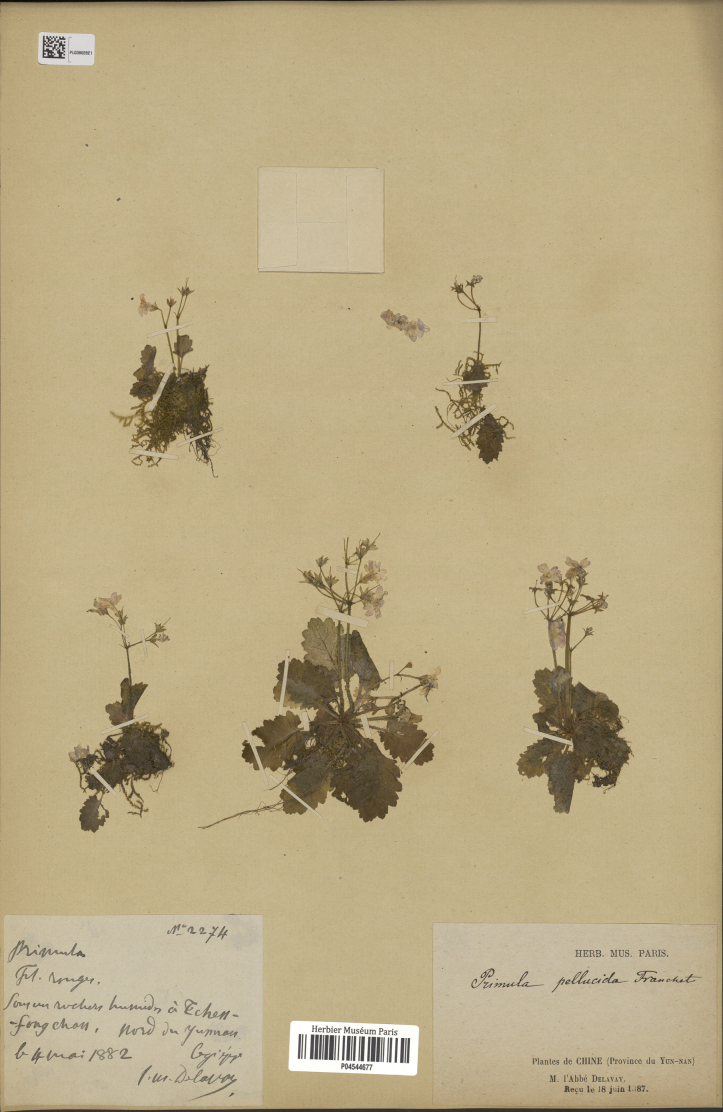
The type specimen of *Primula pellucida* stored in P (*Delavay 2274*, P04544677).

**Figure 6.**
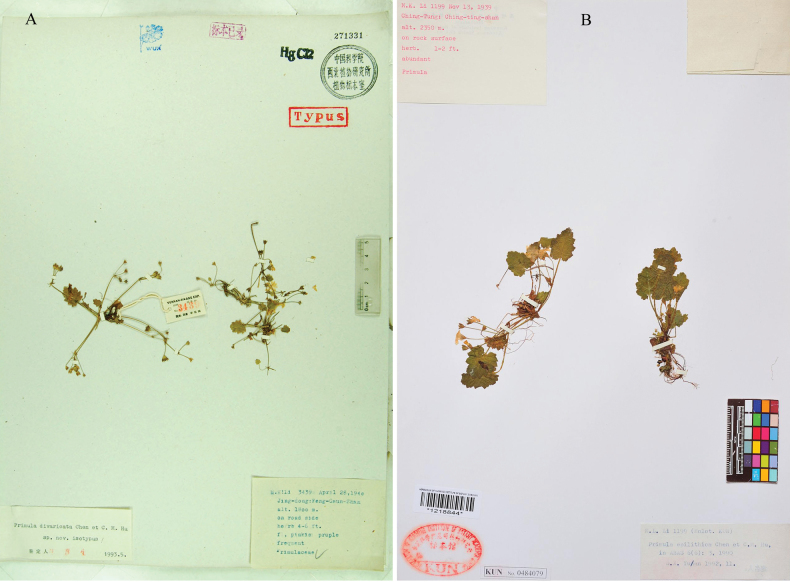
The type specimens of *Primula divaricata* and *P. epilithica*. A. Isotype of *P. divaricata* stored in WUK (*M.K.Li 3439*, 271331); B. Holotype of *P. epilithica* stored in KUN (*M.K.Li 1199*, 1218844).

The new species bears the closest resemblance to *Primula pellucida* (Fig. 5), *P. divaricata* (Fig. 6), and *P. epilithica* (Fig. 6), and we have successfully obtained images of their type specimens. The detailed morphological comparison is shown in Table 1, as well as an identification key to *P. xinjingensis* and its related species.

**Table 1.** Detailed comparisons among *Primula xinjingensis*, *P. pellucida*, *P. divaricata* and *P. epilithica*.

Characters	P. xinjingensis	P. pellucida	P. divaricata	P. epilithica
Habitat	alt. ca. 700 m	alt. ca. 2000 m	alt. 1800–2700 m	alt. 2300–2500 m
Rhizomes	absent	absent	present	present
Calyx				
Calyx shape	campanulate	broadly campanulate	campanulate	campanulate
Lobe shape	lanceolate	triangular to ovate	triangular	ovate-triangular
Lobe apex	acuminate	acute	acute	acute
Lobes in fruit	recurved	recurved	erect	erect
Corolla limb lobes	narrowly cuneate-obovate	obovate	cuneate-obovate	obovate
Pin flowers	style reaching throat	style exserted from corolla tube	style ca. as long as tube	style reaching throat

### ﻿Key to *Primula xinjingensis* and its related species

**Table:** 

1	Inflorescences and abaxial surface of leaves more or less farinose	**2**
–	Plants efarinose throughout	**3**
2	Calyx parted below middle, lobes lanceolate to linear-lanceolate	***P. duclouxii***
–	Calyx parted to the middle, lobes triangular to narrowly triangular	***P. petrocallis***
3	Rhizomes absent, calyx lobes recurved in fruits	**4**
–	Rhizomes present, calyx lobes erect in fruits	**5**
4	Calyx campanulate, and style of pin flowers reaching throat	***P. xinjingensis***
–	Calyx broadly campanulate, and style of pin flowers exserted	***P. pellucida***
5	Scapes with small leaves at apex; calyx 2–3 mm	***P. divaricata***
–	Scapes without leaves at apex; calyx ca. 4 mm	***P. epilithica***

## Supplementary Material

XML Treatment for Primula xinjingensis

## ﻿Additional information

### Use of AI

No use of AI was reported.

### Author ORCIDs

Sheng-Hu Tang https://orcid.org/0000-0002-2168-8559

Ze-Xu Long https://orcid.org/0000-0002-2870-8751

Fang-Wen Li https://orcid.org/0009-0002-6326-5922

### Data availability

All of the data that support the findings of this study are available in the main text.
